# Response of the trial innovation network to the COVID-19 pandemic

**DOI:** 10.1017/cts.2021.782

**Published:** 2021-04-20

**Authors:** Rachel G. Greenberg, Lori Poole, Daniel E. Ford, Daniel Hanley, Harry P. Selker, Karen Lane, J. Michael Dean, Jeri Burr, Paul Harris, Consuelo H. Wilkins, Gordon Bernard, Terri Edwards, Daniel K. Benjamin

**Affiliations:** 1 Duke Clinical Research Institute, Durham, NC, USA; 2 Johns Hopkins University, Baltimore, MD, USA; 3 Tufts University, Boston, MA, USA; 4 University of Utah, Salt Lake City, UT, USA; 5 Vanderbilt University Medical Center, Nashville, TN, USA; 6 Meharry Medical College, Nashville, TN, USA

**Keywords:** Trial innovation network, COVID-19, clinical trial, SARS-CoV-2, CTSA

## Abstract

**Introduction:**

The COVID-19 pandemic prompted the development and implementation of hundreds of clinical trials across the USA. The Trial Innovation Network (TIN), funded by the National Center for Advancing Translational Sciences, was an established clinical research network that pivoted to respond to the pandemic.

**Methods:**

The TIN’s three Trial Innovation Centers, Recruitment Innovation Center, and 66 Clinical and Translational Science Award Hub institutions, collaborated to adapt to the pandemic’s rapidly changing landscape, playing central roles in the planning and execution of pivotal studies addressing COVID-19. Our objective was to summarize the results of these collaborations and lessons learned.

**Results:**

The TIN provided 29 COVID-related consults between March 2020 and December 2020, including 6 trial participation expressions of interest and 8 community engagement studios from the Recruitment Innovation Center. Key lessons learned from these experiences include the benefits of leveraging an established infrastructure, innovations surrounding remote research activities, data harmonization and central safety reviews, and early community engagement and involvement.

**Conclusions:**

Our experience highlighted the benefits and challenges of a multi-institutional approach to clinical research during a pandemic.

## Introduction

Excellence in the development and execution of clinical trials is never more critical than during a pandemic. Clinical trials targeting the pandemic are necessary to demonstrate the safety and efficacy of lifesaving prevention measures, treatments, and vaccines. During a pandemic, the usual delays and inefficiencies that often plague clinical trials should not be tolerated by sponsors or the general public. In the midst of daily case counts and a rising number of widely reported, attributable deaths, delays, and inefficiencies in clinical trials are measured by the lives lost. Thus, operational excellence in the conduct of clinical trials becomes paramount. Simply stated, trials are an even more critically “essential activity” in a pandemic. Since it was established in 2016, the National Center for Advancing Translational Sciences (NCATS)-funded Trial Innovation Network (TIN) has focused on operational innovation and collaboration to support successful multicenter clinical trials within the Clinical and Translational Science Awards (CTSA) Hub consortium, addressing a wide range of diseases and health conditions [1]. The objective of this communication is to describe the response of the TIN to the COVID-19 pandemic and outline the TIN’s structure and function prior to the pandemic, actions taken, and lessons learned from the response.

## TIN Structure and Function

**Fig. 1.**
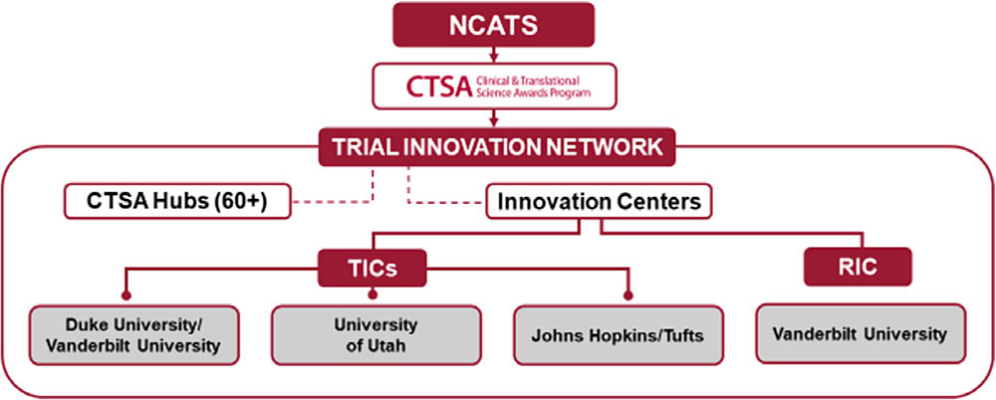
Structure of the Trial Innovation Network (TIN). NCATS: National Center for Advancing Translational Sciences. CTSA: Clinical and Translational Science Award; TIC: Trial Innovation Center; RIC: Recruitment Innovation Center.

The TIN is a collaborative initiative within the NCATS CTSA program that consists of three key partners: the CTSA Program Hubs, the three Trial Innovation Centers (TICs), and the Recruitment Innovation Center (RIC) (Fig. 1). The TIN’s vision is to innovatively address critical roadblocks in clinical research.

Each of the three major TIN components plays a key role in the functions of the TIN. The CTSA Program Hubs are a network of 66 medical research institutions that collaborate locally and regionally to catalyze innovation in training, research tools, and processes. As the key partner of the TIN, the CTSA Program Hubs: (1) encourage faculty and investigators at each Hub institution to generate ideas for trials and studies; (2) provide input before protocols are implemented; (3) help to efficiently identify study sites with sufficient resources and patient populations; (4) support and further develop the essential efforts of their local teams in executing multicenter clinical trials; and (5) create a culture in which key stakeholders play unique and important roles and ultimately work together to build a national system to conduct clinical trials better, faster, and more cost-effectively.

**Fig. 2.**
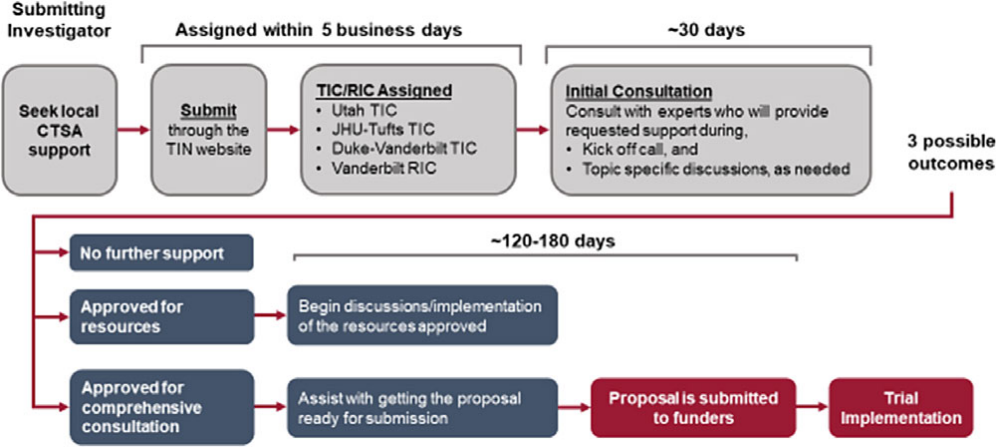
Trial Innovation Network (TIN) proposal submission process. CTSA: Clinical and Translational Science Award; TIC: Trial Innovation Center; RIC: Recruitment Innovation Center; JHU: Johns Hopkins University.

The TIN’s three TICs and RIC are responsible for delivering innovative and high-quality support for the TIN’s clinical trials and projects being carried out at CTSA Hubs [2]. CTSA investigators with an idea for a multicenter clinical trial submit study proposals to the TICs and RIC (Fig. 2). These centers support the investigators via:

- *Initial Consultations*: Initial consultations, which are provided for all submitted proposals, offer a platform for the submitting study team to share the details and vision of their proposal with the TICs, the RIC, and the NCATS. During the consultation, an assigned TIC, the RIC, and NCATS provide input to the submitting team in multiple areas and explore potential collaboration opportunities (Table 1).
  **Table 1.** Trial Innovation Network support available to investigators during initial consultation and resources provided following approval by the proposal assessment team

Initial consultation support	Resources following approval
Protocol design	eConsent
Budget	Single institutional review board (IRB) support
Timelines	Standard agreements
Recruitment	Recruitment and retention plan
Retention	Recruitment materials
Feasibility	Community engagement studio
Grant content/submission	Electronic health record-based cohort assessment
- *Resources*: If the initial consultation indicates that the investigator will benefit from one or more TIN resources, NCATS may approve the provision of these resources (Table 1).
- *Comprehensive Consultations.* Comprehensive consultations function as a collaboration between the submitting study team, the assigned TIC, and the RIC to develop a project in preparation for a funding submission. The TIC and RIC also work with the study’s PI to develop and incorporate operational innovation into the proposal.
- *Study Implementation*. The assigned TIC may assist in study implementation after a comprehensive consultation has been completed and the study has received funding. The total scope of implementation for the TIC varies based on project needs and collaborator preferences.

Since its launch in 2016, the TICs and RIC have performed 301 initial consultations and 39 comprehensive consultations. Discrete approved resources have been provided to 99 proposals, and the TIN has implemented 14 studies and five pilot trials. Prior to the pandemic, the TIN performed an average of five initial consultations per month. The TICs and RIC routinely assess satisfaction from investigators who receive consultations; among 18 investigators not affiliated with TIC/RIC institutions who responded to a survey following consultation during the pandemic, 94% reported that they would return or recommend the TIN to their colleagues for initial consultation. To protect confidentiality of investigators, responses from COVID-related consultations could not be differentiated from other consultations not related to COVID-19.

## How did the TIN adapt or change during the COVID-19 pandemic?

As the COVID-19 pandemic spread across the USA, there arose a critical need for clinical trials targeting both preexisting and newly developed therapeutics. Approximately 250 interventional clinical trials were posted on the clinicaltrials.gov website by May 30, 2020 [3]. The TICs and RIC performed 29 COVID-related consultations between March 2020 and October 2020 (Table 2). The TICs and RIC encountered several challenges related to these COVID-related consultation requests:

1. Even though the number of patients affected with COVID-19 was growing across the USA during the spring and summer of 2020, the number of hospitalized patients available to participate in clinical trials was small compared to the number of research questions.
2. With the rapidly changing landscape of COVID-19 hotspots, identifying ideal clinical trial sites was particularly challenging. Cohort assessments based on the electronic health record (EHR) were not always reliable in projecting which potential sites would have the greatest numbers of eligible participants. This number changed quickly at sites; and at some sites was in near-constant flux.
3. Many potential clinical trial sites struggled to identify efficient methods to execute the logistics of clinical trial site activation and execution, including contracting, site location, assessment of available staff, consenting participants, and the safe implementation of study procedures. This was particularly true for outpatient COVID positive participants.
4. The burden of COVID-19 has disproportionately impacted Black, Hispanic/Latino, Indigenous people, and individuals with limited English proficiency (LEP) [4–7]. Historically, these populations have been underrepresented in clinical trials; thus, engaging them during a pandemic was even more challenging.

**Table 2.** Select COVID-related consultations provided by the Trial Innovation Network (TIN)

Date submitted	Project description	Institution	TIN support provided
4/8/2020	Randomized, placebo-controlled Phase II clinical trial to evaluate the safety and efficacy of a modified tetracycline for the treatment of COVID-19 patients	Stony Brook University	-High-level statistical consultation and planning
			-Regulatory advice regarding what would be needed for the Investigational New Drug application
4/14/2020	Prospective cohort study of patients with asthma to evaluate the effects of COVID-19	University of North Carolina	Single IRB support consultation
4/14/2020	Prospective, fully virtual cohort study of mothers and infants with exposure to COVID-19	Medical University of South Carolina	-Advice regarding study design, budget, and projected timelines
4/14/2020	Randomized, placebo-controlled Phase II clinical trial of valproic acid in patients with symptomatic COVID-19	The University of Texas Health Science Center at San Antonio	-Comprehensive consultation including protocol development, biostatistics, and proposal development.
			-Recruitment feasibility assessment
			-Community engagement studio
4/15/2020	Randomized, placebo-controlled Phase II clinical trial to evaluate the safety and efficacy of ramipril for the treatment of COVID-19 patients	University of California, San Diego	-Recruitment and retention planning
			-Assessment of study feasibility
4/21/2020	Direct-to-family observational, prospective cohort study of children exposed to COVID-19	Penn State Milton S. Hershey Medical Center	-Comprehensive consultation with advice on study design, study budget, data solutions, single IRB, recruitment, and retention planning
5/22/2020	Randomized, placebo-controlled trial of prophylactic probiotics to prevent COVID-19 transmission in patients undergoing surgery	University of North Carolina	-Comprehensive consultation with advice on site management and monitoring, study budget, and statistical design. Recommended ePRO via REDCap
6/3/2020	Randomized, placebo-controlled Phase II clinical trial to evaluate the safety and efficacy of inhaled salmeterol for the treatment of COVID-19 patients	Medical College of Wisconsin	-Comprehensive consultation with advice on IND/regulatory approach, study design, study budget, and recruitment and retention planning
6/9/2020	Platform trial to validate point-of-care devices for the detection of COVID-19	University of Massachusetts Medical School, Worcester	-Advice regarding study design, recruitment and retention planning, and assessment of study feasibility
6/21/2020	Randomized, placebo-controlled, Phase II Bayesian adaptive clinical trial of vadadustat for the prevention and treatment of ARDS in hospitalized patients with COVID-19	University of Texas Health Science Center at Houston	-Advice regarding study design, and adaptive design. Discussion of statistical plan in the context of how COVID trials are looking at endpoints
6/19/2020	ACTIV-1: Randomized, placebo-controlled Phase II clinical trial to evaluate the safety and efficacy of a 3 immunomodulators for the treatment of COVID-19 patients	Washington University	-EHR-based cohort assessment, expression of interest, statistical planning.
			-Development of study specific recruitment materials
9/25/2020	Prospective cohort study to evaluate long-term brain effects of COVID-19 via imaging	University of Minnesota Twin Cities	-Comprehensive consultation supporting rapid proposal development.
			-Community engagement studio
			-Recruitment materials
9/29/2020	ACTIV-2: Adaptive platform treatment trial for outpatients with COVID-19	National Institute of Allergy and Infectious Diseases	-EHR-based cohort assessment, expression of interest
10/22/2020	Non-inferiority trial to comparing fracture liaison service via in-person vs telehealth in the COVID-19 era	The University of Alabama at Birmingham	-Resources including recruitment feasibility assessment, single IRB, and standard agreements
12/9/2020	Randomized, placebo-controlled Phase II clinical trial to evaluate the safety and efficacy of a repurposed drug given via direct lung instillation for the treatment of COVID-19 patients	University of Minnesota Twin Cities	-Advice regarding study design, recruitment and retention planning, assessment of study feasibility, and adaptive design

IRB, institutional review board; HER, electronic health record; NIAID, National Institute of Allergy and Infectious Diseases; ePRO, electronic patient reported outcome; IND, investigational new drug.

The TICs and RIC adapted to address these challenges in several ways. The TICs and RIC developed processes to triage the increased number of COVID-related proposals. Discussions of each potential study focused early on trial feasibility and the ways in which the trial would complement or compete with other trials that were already being performed. Given the changing geographical landscape of the focus of the infection, the consulting teams advised investigators to place EHR-based cohort assessments in context for the selection of clinical sites.

The TIN took numerous steps to address the logistical challenges of conducting clinical research during a pandemic. The challenges of in-person participant recruitment and follow-up offered an ideal opportunity for innovation using direct-to-patient, remote methodologies. For example, one TIC and the RIC assisted faculty at Medical University of South Carolina in the development of a fully remote, observational cohort study that aimed to examine the outcomes of infants who were exposed to COVID-19 by enrolling mother-infant pairs. The study engaged clinical sites to advertise participation to potential participants, but the participants self-enrolled via a study website using electronic consent. All follow-up occurred remotely via questionnaires and remote interviews. The TICs and RIC also helped multiple trials transition to electronic consenting procedures or transition from being in-person to becoming fully virtual.

The RIC leveraged one of its flagship programs for connecting with the community at large: community engagement studios (CE studios), a model for gathering community member input on research [8]. The CE studio elicits project-specific input from community stakeholders typically during an in-person, moderated roundtable. During the pandemic, the CE studios successfully shifted to a virtual format and as a result, have engaged individuals from diverse communities across the country in recruitment and retention planning.

## What Were Best Practices or Lessons Learned?

The most important advantage the TIN had in its response to the COVID-19 pandemic was its pre-established infrastructure and the fact that the TIN’s mission was not restricted to supporting one disease. This infrastructure was critical because: (1) communication lines were already in place among a vast network of academic institutions capable of implementing clinical trials; (2) there existed a central body and mechanism for the development and review of proposals and triage facilitation and prioritization; (3) there were experienced coordinating centers familiar with rapid start-up and site activation. The TIN had already leveraged this infrastructure to operationalize coordination of the Helping to End Addiction Long-term (HEAL) Pain Management Effectiveness Research Network (ERN). This multisite research cooperative is leading and implementing multiple clinical trials to address the opioid epidemic by testing alternative strategies for the prevention and treatment of acute and chronic pain. Pivoting from the opioid epidemic to the COVID-19 pandemic allowed the TIN to leverage existing tools and strategies but also highlighted some challenges unique to the pandemic. For example, the TICs and RIC responded to a consultation from Washington University to engage in the ACTIV-1 trial, a phase 3 clinical trial that will enroll approximately 2,000 adults in the USA and Latin America hospitalized with moderate to severe cases of COVID-19 [4]. The consultation proposal requested an EHR-based cohort assessment of the COVID-19 population, as well as the identification of interested principal investigators within the CTSA network and their affiliates. Building on previous experience, the assigned TIC and the RIC coordinated an investigator-led webinar on the ACTIV-1 protocol six days following the initial request. Responses to the outreach were received quickly, with the first response received the day of the webinar and an average response time of nine days. Once interested PIs were identified, study-specific feasibility surveys were sent to 56 interested sites (16 days after initial request). Sites responded within two weeks. This early identification of interested sites and their completion of the survey facilitated site start-up following the selection of the coordinating center. Once contracts were sent to the selected sites, the initiation visit of the first site was held one week later and the first site was activated two weeks later. The first participant was enrolled one day after the activation of the first site.

However, multiple challenges were encountered following the speed of initial site activation. Some challenges were specific to the pandemic. These challenges included a reduced availability of study coordinators and the presence of other studies competing for limited resources, such as information technology resources for electronic medical record builds and review of contracts. Some challenges existed independent of the pandemic itself but were especially impactful due to the pandemic-related need for speedy trial completion. For example, many study sites required start-up activities (such as contract review or IRB approval) to occur sequentially rather than in parallel. Efficient agreement on contract language was extremely variable between sites. Some of this variation was attributed to unique aspects of flow down language placed in the master contract by the sponsor. In addition, even in the setting of single IRB review, many local IRBs require levels of additional review that delay activation. In fact, “local context review” results in considerable potential delay of activation in the single IRB review era. Site investigators often have little control over these issues, and the coordinating center lacks the ability to accelerate these processes.

Because of its robust infrastructure, the TIN became a central reviewing body of a large number of COVID-related trial proposals, many of which were otherwise funded by either industry or separate funding agency sponsors and would not have had central oversight. This unique vantage point allowed the TIN to learn from each trial, incorporating successful strategies when applicable and foreseeing challenges with certain protocols. In large trials, for example, the TIN recognized the need for detailed and frequently planned interim analyses so that critical data such as baseline severity, comorbidities, and critical event rates could be reviewed at real-time intervals during the pandemic.

### Combining efforts across CTSA Hubs

Two TIN programs are in ongoing efforts to leverage cooperative activity across the CTSA Hubs by pooling individual participant data from small trials to produce an individual patient analysis of data across trials. A similar exercise occurring in real time is also being performed to compare, contrast, and combine safety data from multiple trials in real time to increase safety communication among autonomous trial DSMBs during the accrual period of trials.

Piloting efficacy data pooling has focused on several inpatient trials that ended accrual in late spring because the pandemic slowed, and several peer-reviewed signals suggested a small or no benefit with the possibility of increased safety events in the active exposure arm. This effort has involved comparing individual trial data dictionaries, harmonizing data elements, developing cross-trial consensus on the prioritization of clinical hypotheses to analyze, development of a consensus on the statistical analysis plan, organizing an authorship and publication plan, and combining multiple limited data sets with protection of protected health information.

The TIN DSMB Coordination (DSMBc) effort sought to create visualization and data reporting tools to enable the synchronized review of multiple studies by a single DSMB focused on a specific therapy using harmonized reports (hReports) [9]. The DSMBc organizing principle in which many clinical trials have several analytical and reporting methods in common (e.g., patient accrual reporting) and in which higher-order functions are utilized to implement these procedures in a common way. The DSMBc effort was piloted using four randomized convalescent plasma controlled trials. Using analytical tools, the DSMBc biostatisticians mapped key risk and outcome variables between the case report forms. This allowed requests for information from across studies to be simplified and expedited when it was time for any trial DSMB to meet. Standardization of data reporting across studies enabled cross-study comparisons to allow individual trial DSMBs to investigate safety and efficacy signals from participating studies to inform their decision making.

### Supporting Dissemination

The TIN sponsored numerous activities during the pandemic that supported dissemination of research-related activities. Examples include over 13 public-access TIN hosted Webinars and Open Forums related to COVID-19 related research activities and resources. One of these webinars held in March 2020 and attended by over 400 participants targeted self-service tools for eConsent, 21CFR Part-11 validation, and best-practice videos related to development and support of eConsent methods. Based on consultations performed during the pandemic, the RIC developed a COVID-19 Recruitment and Retention Toolkit which is publicly available. The TIN also supports a persistent messaging hub for approximately 200 local CTSA recruitment and retention experts. Communication on this messaging platform may include any topic related to participant recruitment and retention, but COVID-19 related discussions have been popular during the pandemic.

## Which of the Changes Might Apply to non-COVID Research or Should Become Permanent?

Many of the TIN’s adaptations during the COVID-19 pandemic could be applied to non-COVID research. Remote research has multiple possible benefits, including added convenience for study participants, improved retention of participants, and reduced costs. The use of electronic consent should be considered for any study. The direct-to-patient approach to clinical research also allows for the recruitment of participants from rural and other communities that may not have access to the classic academic clinical research site.

**Fig. 3.**
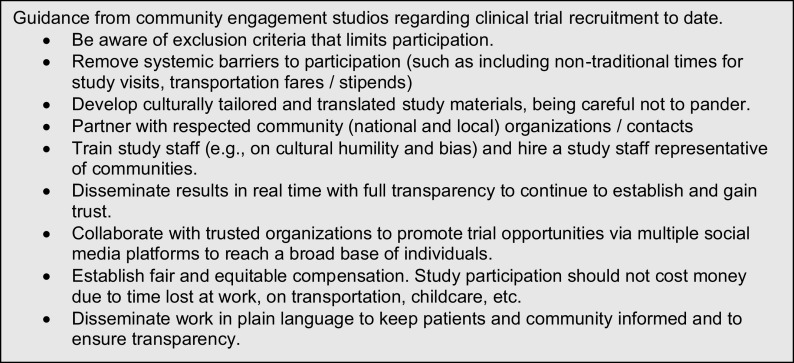
Clinical trial recruitment best practices coming from community engagement studios.

One important lesson from the COVID-19 pandemic is the need for the early engagement of the lay community. The alarming number of cases of morbidity and mortality related to COVID-19 in vulnerable populations makes the enrollment of affected groups in clinical trials a priority. The rapid pace of research during a pandemic can challenge principles of engagement, which focus on engendering trust and gaining buy-ins from communities. However, many CTSA Hubs had already built robust community engagement programs, which encouraged the community to understand research needs and meet these challenges. The CTSA program’s commitment to community engagement provided research teams at the Hubs the ability to consult with community research advisory groups who were already familiar with the ways in which research teams and community groups could work together to support clinical trials. The RIC team quickly shifted to virtual CE studios and completed eight CE studios focused on COVID-19 projects, with important lessons learned on conducting studies during the pandemic (Fig. 3). In addition, the RIC leveraged its long-standing community advisory board (CAB), which included individuals representing Black, Hispanic/Latino, Asian, American-Indian, and rural communities from across the nation, who have provided formative and insightful feedback on barriers to COVID-19 research participation. These efforts again solidified the need for independent, authentic feedback from community members and were utilized in multiple COVID-19 studies.

The COVID-19 pandemic also offered a unique opportunity for building other new relationships with the community that we hope will become permanent. One example of such a relationship was proposed to the TIN as an initial consultation. The project, now funded by NICHD, links school leaders with CTSA sites to interpret emerging scientific evidence to keep children, teachers, and the community healthy and safe during the COVID-19 pandemic. The long-range vision for community engagement is that these relationships will become a foundation for further outreach and research that will positively impact public health.

## Conclusion: What Recommendations Would the TIN Have for Being Prepared for Future Pandemics?

While the COVID-19 pandemic is ongoing at the time of this communication, thus limiting definitive conclusions and comprehensive assessment of the effectiveness of the TIN’s pandemic-related activities, the TIN’s early experiences have yielded several recommendations that might be applied to future pandemics. The following recommendations were produced as a collaborative effort among the leadership of the TICs and RIC, including multiple faculty members who also lead CTSA Hubs.

## Recommendations

1. **Emphasize** the essential nature of research during any pandemic.
2. The nation should invest in **multisite clinical trial infrastructure** that is prepared to respond to new health threats and can be quickly leveraged to develop and implement the most pivotal clinical trials. An efficient clinical trial network should not be freshly launched during a new pandemic; it should be well-practiced with a track record of success *prior to* the pandemic.
3. **Remote activities** and a **direct-to-participant approach** can facilitate research at times when in-person, site-based activities are neither feasible nor preferred.
4. **Early recognition** of the limits of non-pandemic clinical trial site assessment tools (e.g., traditional data warehouse-based, EHR-based cohort assessments) and pivoting to new methods (relying on site-specific methods already rendering COVID-19 census reports), can accelerate timelines and set realistic expectations for enrollment.
5. **Data harmonization** should be an early focus. Early guidance regarding preferred definitions for primary and secondary outcomes would streamline clinical trial development and substantially improve the ability to interpret results across studies. Consortium level master protocols could enhance the cross trial harmony of subsequent data.
6. When possible, combining trial data and **data safety monitoring boards** increases the relevance of smaller studies and the probability of observing important safety signals for therapeutics that are likely to become widely used.
7. **Community involvement** in study development is critical in building trust in scientific and medical activities and can have a long-lasting, positive impact on public health. Rapidly engaging marginalized communities during a pandemic requires community engagement infrastructure and prior investment in mutually beneficial community-academic partnerships.
